# Standard Flow Liquid Chromatography for Shotgun Proteomics in Bioenergy Research

**DOI:** 10.3389/fbioe.2015.00044

**Published:** 2015-04-01

**Authors:** Susana M. González Fernández-Niño, A. Michelle Smith-Moritz, Leanne Jade G. Chan, Paul D. Adams, Joshua L. Heazlewood, Christopher J. Petzold

**Affiliations:** ^1^Joint BioEnergy Institute and Physical Biosciences Division, Lawrence Berkeley National Laboratory, Berkeley, CA, USA; ^2^Department of Bioengineering, University of California Berkeley, Berkeley, CA, USA; ^3^Australian Research Council Centre of Excellence in Plant Cell Walls, School of Botany, The University of Melbourne, Melbourne, VIC, Australia

**Keywords:** proteomics, standard flow chromatography, biofuels, mass spectrometry

## Abstract

Over the past 10 years, the bioenergy field has realized significant achievements that have encouraged many follow on efforts centered on biosynthetic production of fuel-like compounds. Key to the success of these efforts has been transformational developments in feedstock characterization and metabolic engineering of biofuel-producing microbes. Lagging far behind these advancements are analytical methods to characterize and quantify systems of interest to the bioenergy field. In particular, the utilization of proteomics, while valuable for identifying novel enzymes and diagnosing problems associated with biofuel-producing microbes, is limited by a lack of robustness and limited throughput. Nano-flow liquid chromatography coupled to high-mass accuracy, high-resolution mass spectrometers has become the dominant approach for the analysis of complex proteomic samples, yet such assays still require dedicated experts for data acquisition, analysis, and instrument upkeep. The recent adoption of standard flow chromatography (ca. 0.5 mL/min) for targeted proteomics has highlighted the robust nature and increased throughput of this approach for sample analysis. Consequently, we assessed the applicability of standard flow liquid chromatography for shotgun proteomics using samples from *Escherichia coli* and *Arabidopsis thaliana*, organisms commonly used as model systems for lignocellulosic biofuels research. Employing 120 min gradients with standard flow chromatography, we were able to routinely identify nearly 800 proteins from *E. coli* samples; while for samples from *Arabidopsis*, over 1,000 proteins could be reliably identified. An examination of identified peptides indicated that the method was suitable for reproducible applications in shotgun proteomics. Standard flow liquid chromatography for shotgun proteomics provides a robust approach for the analysis of complex samples. To the best of our knowledge, this study represents the first attempt to validate the standard flow approach for shotgun proteomics.

## Introduction

Advances in biofuels research focusing on feedstock characterization and engineering (Persil-Cetinkol et al., 2012; DeMartini et al., 2013; Shen et al., 2013; Eudes et al., 2014) as well as the genetic manipulation of microbes (Alper et al., 2006; Tyo et al., 2007; Keasling, 2008; Lee et al., 2008) have progressed significantly in the last few years. Unfortunately, analytical capabilities required to efficiently monitor and assess these changes are lagging. Furthermore, many modern bioanalytical techniques are focused toward medical and health related research, which have significantly different priorities and requirements for success. Most biotechnology research challenges are not constrained by sensitivity or resolution of an assay, rather they depend on accurate identification and quantitation of target molecules for a large number of samples. Consequently, an important component for biotechnological research is sample throughput supported by a robust analytical platform. Recent advances in proteomics and metabolomics have focused on liquid chromatography-mass spectrometry (LC-MS) methods by increasing their sensitivity to aid discovery-based research efforts. This is most evident with the development of nano-LC couple to high-resolution mass spectrometers; yet, this technology is yet to mature into a robust platform capable of consistently analyzing hundreds of samples per week. Consequently, alternate technologies capable of answering the questions necessary for biotechnology progress are needed. Recently, we published a high throughput targeted proteomic toolkit based on standard flow chromatography coupled to mass spectrometry (MS) to help address these issues for *Escherichia coli*; however, a significant amount of methods development was necessary. This work prompted us to assess the utility of standard flow liquid chromatography (LC) for shotgun proteomic methods related to biotechnology.

The discipline of proteomics has been dominated by nano-flow LC coupled to MS since its early development over 20 years ago (Emmett and Caprioli, 1994; Gatlin et al., 1998). The adoption of nano-flow LC for protein identification was driven by the substantial increases in sensitivity and detection capabilities of nano-flow (ca. 500 nL/min) over capillary (ca. 50 μL/min) and standard flow (ca. 0.5 mL/min) chromatography. Typically, shotgun proteomic studies utilize nano-flow chromatography methods due to limited amounts of sample and to obtain optimal ionization efficiency for sensitive detection of peptides but at the cost of ease of use and system robustness (Gapeev et al., 2009). These issues are particularly problematic for biotechnology research that depends heavily on high sample throughput. Recently, ultra-high performance LC coupled to triple quadrupole mass spectrometers has been shown to yield comparable sensitivity and better analytical metrics (coefficient of variation, dynamic range) than nano-flow LC for MRM-based analysis of biomarker proteins when the amount of sample is adjusted to the column size (Percy et al., 2012). Additionally, the well-established robust operation of standard flow chromatography makes this instrumentation attractive for applications that rely on high sample throughput, consistent results, and less system downtime (Swartz, 2005). For applications where sample abundance is not limited, one of the greatest concerns with ultra-high pressure liquid chromatography-mass spectrometry (UHPLC-MS) workflows is the loss of sensitivity, relative to nano-LC-MS workflows, leading to datasets of insufficient depth to answer questions of interest.

The adoption of a UHPLC-MS workflow for sample delivery requires both efficient ionization and instrument speed to handle both the sample delivery rate and reduced elution times for peptides. The past decade has seen significant developments and advances in instrumentation associated with proteomics-based MS. Advances in reversed phase C18 chromatography columns yield greater separation efficiency and more stable retention times. The current generation of instrumentation is faster, more sensitive, and is better able to deal with the dynamic range inherent in biological samples. Moreover, the development and adoption of off-axis nebulizers for sample delivery when using electrospray ionization significantly reduces contamination of capillaries and skimmers (Banerjee and Mazumdar, 2012). Collectively, these improvements have enabled the development of a UHPLC-MS workflow for MRM-based analysis of biomarker proteins (Percy et al., 2012). Consequently, we were interested in assessing the capacity of this workflow in shotgun proteomic experiments. The work described here details the results of an analysis of a prokaryote (*E. coli*) and an eukaryote (*Arabidopsis thaliana*) whole cell proteomes on an Agilent UHPLC-QTOFMS system, but would be generally applicable for any current generation of tandem mass spectrometer being utilized for shotgun proteomics.

## Materials and Methods

### Protein extraction

Protein was extracted using standard techniques with analytical reagents where suitable. For *E. coli* DH5α samples, cell lysis and protein precipitation was accomplished using a chloroform/methanol precipitation. A 100 μL aliquot of cells was transferred to a 1.7 mL tube, followed by the addition of 400 μL of methanol, 100 μL of chloroform, and 300 μL of water, with mixing by vortex after each addition. Following centrifugation at 21,000 × *g* for phase separation, the methanol and water layer was removed and 300 μL of methanol was added. The tube was briefly vortexed to dislodge the protein pellet, then centrifuged at 21,000 × *g* for 2 min. The chloroform and methanol layer was removed and the protein pellet was dried for 5 min in a vacuum concentrator. The protein pellet was re-suspended in 100 mM (NH_4_)HCO_3_ with 20% methanol, reduced with 5 mM TCEP [Tris(2-carboxyethyl)phosphine hydrochloride] for 30 min at room temperature, treated with 10 mM iodoacetamide (IAA) for 30 min in the dark at room temperature, and digested with trypsin (1:50 w/w) overnight at 37°C. Aliquots of 40 μg were taken for analysis by LC-MS/MS. For *A. thaliana* (L.) Heynh. (ecotype Landsberg erecta), protein was extracted from a previously described heterotrophic cell culture (Ito et al., 2011). A total of 1 g plant material (fresh weight) was used for the isolation of total protein. The plant material was harvested and frozen with liquid nitrogen in an Eppendorf tube with two small steel balls. The protein extraction was performed by the addition of 0.4 mL of fresh disruption buffer [125 mM Tris-HCl, 7% (w/v) SDS, and 10% β-mercaptoethanol], followed by vortex for 10 min at room temperature. The samples were centrifuged at 10,000 × *g* for 5 min at 4°C and the supernatant separated into two 2 mL tubes. Samples were further extracted in 800 μL methanol and mixed, then 200 μL chloroform added and mixed, and finally 500 μL of ddH_2_O added and vortexed (30 sec each time). Samples were centrifuged for 5 min at 10,000 × *g* at 4°C, the aqueous phase removed, and 500 μL of methanol added. Samples were vortexed for 30 s and centrifuged for 10 min at 9,000 × *g* at 4°C. The supernatant was discarded and the pellet air-dried. The dried pellet was suspended in 200 μL of re-suspension buffer [3M urea, 50 mM (NH_4_)HCO_3_, and 5 mM dithiothreitol, pH 8], and incubated with IAA at a final concentration 10 mM for 30 min in the dark. Prior to analysis by MS, 40 μg of extracted protein was digested with trypsin (1:10 w/w) overnight at 37°C. Peptides were desalted using C18 Micro SpinColumns (Harvard Apparatus) as previously outlined (Parsons et al., 2013). Eluted peptides were re-suspended in 2% acetonitrile, 0.1% formic acid prior to analysis by LC-MS/MS.

### Standard flow mass spectrometry

All samples were analyzed on an Agilent 6550 iFunnel Q-TOF mass spectrometer (Agilent Technologies) coupled to an Agilent 1290 UHPLC system. Peptide samples were loaded onto a Sigma–Aldrich Ascentis Peptides ES-C18 column (2.1 mm × 100 mm, 2.7 μm particle size, operated at 60°C) via an Infinity Autosampler (Agilent Technologies) with Buffer A (2% acetonitrile, 0.1% formic acid) flowing at 0.400 mL/min. Peptides were eluted into the mass spectrometer via a gradient with initial starting condition of 5% buffer B (98% acetonitrile, 0.1% formic acid). For analysis of all samples, buffer B was increased to 35% over 120 min. Buffer B was then increased to 50% over 5 min, then up to 90% over 1 min, and held for 7 min at a flow rate of 0.6 mL/min, followed by a ramp back down to 5% B over 1 min where it was held for 6 min to re-equilibrate the column to original conditions. Peptides were introduced to the mass spectrometer from the LC by using a Jet Stream source (Agilent Technologies) operating in positive-ion mode (3,500 V). Source parameters employed gas temp (250°C), drying gas (14 L/min), nebulizer (35 psig), sheath gas temp (250°C), sheath gas flow (11 L/min), VCap (3,500 V), fragmentor (180 V), OCT 1 RF Vpp (750 V). The data were acquired with Agilent MassHunter Workstation Software, LC/MS Data Acquisition B.05.00 (Build 5.0.5042.2) operating in Auto MS/MS mode whereby the 20 most intense ions (charge states, 2–5) within 300–1,400 m/z mass range above a threshold of 1,500 counts were selected for MS/MS analysis. MS/MS spectra (100–1,700 m/z) were collected with the quadrupole set to “Medium” resolution and were acquired until 45,000 total counts were collected or for a maximum accumulation time of 333 ms. Former parent ions were excluded for 0.1 min following MS/MS acquisition.

### LC-MS/MS data analysis and integration

The acquired data were exported as .mgf files using the Export as MGF function of the MassHunter Workstation Software, Qualitative Analysis (Version B.05.00 Build 5.0.519.13 Service Pack 1, Agilent Technologies) using the following settings: peak Filters (MS/MS), the absolute height (≥ 20 counts), relative height (≥ 0.100% of largest peak), maximum number of peaks (300) by height; for charge state (MS/MS), the peak spacing tolerance (0.0025 m/z plus 7.0 ppm), isotope model (peptides), charge state limit assigned to (5) maximum. Resultant data files were interrogated with the Mascot search engine version 2.3.02 (Matrix Science) with a peptide tolerance of ±50 ppm and MS/MS tolerance of ±0.1 Da; fixed modifications Carbamidomethyl (C); variable modifications Oxidation (M); up to one missed cleavage for trypsin; peptide charge 2+, 3+, and 4+; and the instrument type was set to ESI-QUAD-TOF. Searches were performed against either an *E. coli* (strain K12) dataset obtained from UniProt (Magrane and Consortium, 2011) or the latest release of the *A. thaliana* dataset comprising TAIR10 obtained from The Arabidopsis Information Resource (Lamesch et al., 2012). Both databases incorporated proteins comprising the common Repository of Adventitious Proteins (cRAP v2012.01.01 from The Global Proteome Machine). The *E. coli* database comprised 4,429 sequences (1,398,775 residues) while the *Arabidopsis* database comprised 35,508 sequences (14,522,421 residues). Protein and peptide matches identified after interrogation of MS/MS data by Mascot were filtered and validated using Scaffold v4.3.0 (Proteome Software Inc.). Peptide identifications were accepted if they could be established at >95.0% probability by the Peptide Prophet algorithm (Keller et al., 2002) with Scaffold delta-mass correction. Protein identifications were accepted if they could be established at >95.0% probability and contained at least 1 identified peptide (at 95% and greater). Protein probabilities were assigned by the Protein Prophet algorithm (Nesvizhskii et al., 2003). This resulted in false discovery rates of 0.9 (*E. coli*) and 0.3% (*Arabidopsis*) for protein and 0.37 (*E. coli)* and 1.66% (*Arabidopsis*) for peptides. Proteins that contained similar peptides and could not be differentiated based on MS/MS analysis alone were grouped to satisfy the principles of parsimony.

### Statistical data analysis

Statistical analysis was performed using the statistical toolbox in MATLAB v2009b (Mathworks). The following analysis was performed on data from both *Arabidopsis* and *E. coli*. After data filtering using Scaffold v4.3.0 (outlined above), peptide data were exported as .csv files and only peptides derived from the same protein identified across all replicates were considered. The Mascot ions score and total ion current values were only used for the best matching peptide (based on ion score) in each replicate. The total spectrum count for each peptide from each replicate was calculated manually. The coefficient of variation (CV: SD/mean) of total spectrum counts, Mascot ions score, and total ion current for each of the common peptides across the replicates was calculated to determine the variation across the technical replicates. The histograms of the CV were plotted to determine distribution and general extreme value fit algorithm was used to determine fit and mean of the distribution. For principal component analysis (PCA), the total spectrum count, Mascot ions score, and total ion current for all the common peptides of each technical replicate were mean centered. PCA was done by eigenvalue decomposition of the data covariance, resulting in a set of linearly uncorrelated variables. The principal component scores were then plotted to identify groupings.

## Results and Discussion

The initial setup and tuning of the standard flow LC-MS/MS parameters was undertaken using 20 fmol aliquots of trypsin digested bovine serum albumin (BSA). The system settings were deemed adequate once identification of BSA was comparable (i.e., unique peptides, total peptides, and coverage) to that achieved using previously benchmarked nano-flow LC-MS/MS approaches on a variety of instruments over the past 5 years.

### Application of standard flow LC-MS/MS with prokaryotic samples

**Figure 1:**
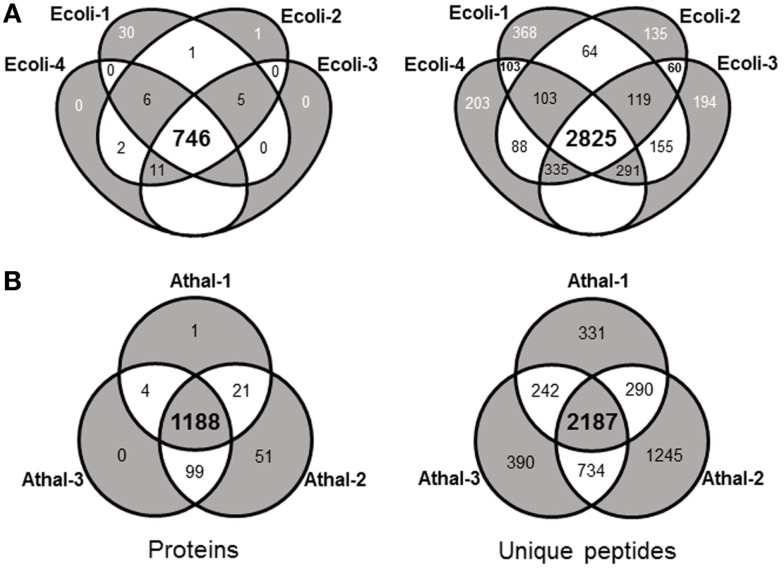
**Venn diagrams highlighting the shared matched proteins and unique peptides between technical replicates from (A) *E. coli* and (B) *Arabidopsis* total protein samples analyzed with the standard flow LC-MS/MS approach**.

Initial experiments were performed on *E. coli*, a well-characterized organism with minimal proteomic complexity. The *E.coli* genome of the widely utilized laboratory strain K-12 was completed nearly 20 years ago (Blattner et al., 1997). The genome has undergone multiple revisions and is estimated to codes for over 4,300 proteins (Magrane and Consortium, 2011). Shake flasks of *E. coli* DH5α cultures were grown aerobically at 37°C on Luria Broth (LB) medium supplemented with 1% glucose. Total protein was extracted and digested overnight with trypsin at 37°C. The analysis procedure for *E. coli* samples was developed to take advantage of the speed and robustness of a standard flow analysis and as such, a method was developed that incorporated a 120 min gradient. A total of four biological replicates each equivalent to 40 μg of peptide were analyzed by a standard flow UHPLC-QTOFMS operating with typical shotgun proteomics data acquisition parameters. The samples yielded on average 31,938 ± 989 (SE) MS/MS spectra (Table 1). Each of these MS/MS datasets was used to query the *E. coli* (K12) protein database from UniProt (Magrane and Consortium, 2011) using the Mascot search engine (Matrix Science) with resultant protein matches filtered using Scaffold (Proteome Software). In total, 802 proteins were identified from the four replicates with an average of 786 ± 4.5 (SE) per sample (Table 1 and Table S1 in Supplementary Material). This represents about 20% of the protein coding capacity of *E.coli*. On average, 14,639 ± 484 (SE) MS/MS spectra were successfully matched to a peptide, corresponding to around 46% of the total queries for each sample (Table 1). This would be regarded as a high conversion rate indicating that the acquired MS/MS was of sufficient quality to confidently assign nearly 50% of the queries using the approach. The consistency of the standard flow approach was highlighted by the total number of proteins assigned in each sample. A total of 768 proteins were identified in three out of four replicates, while 746 proteins (93%) were identified collectively in all four samples. This compares well to the total number of proteins identified in all samples (802). Few proteins were identified uniquely in a single replicate except for replicate Ecoli-1, where 30 unique proteins were identified (Figure 1A). Consequently, we sought to examine whether this setup was adequately dealing with the sample complexity given the flow rate and an approximate peptide elution time of around 6 s (Figure S1 in Supplementary Material). Nearly 75% of the matched MS/MS spectra were redundant, with an average of 4,009 unique peptides per sample (Table 1). Only 2,825 unique spectra (54%) were shared between all four samples; however, the new unique peptides (2,393) accounted for only 56 new proteins (Figure 1A). The approach consistently identifies the major proteins in a sample and a majority of unique spectra assigned between replicates are derived from previously identified proteins. This indicates that the QTOFMS has the capacity to handle the standard flow UHPLC setup in shotgun mode even at these higher flow rates and reduced peptide elution times.

**Table 1:** **Values obtained from *E. coli* (Ec) and *Arabidopsis* (At) samples analyzed using the standard flow technique**.

	Ec 1	Ec 2	Ec 3	Ec 4	At 1	At 2	At 3
Queries	31,452	29,348	33,638	33,315	26,135	37,388	28,203
Spectra	14,677	13,265	15,439	15,173	6,038	9,712	6,846
Spectra matched (%)	47	45	46	46	23	26	24
Unique peptides	4,028	3,729	4,154	4,123	3,050	6,643	3,553
Total proteins	788	772	791	791	1,214	1,359	1,291

### Application of standard flow LC-MS/MS to a complex eukaryotic sample

Next, we were interested in assessing the suitability of the standard flow proteomics platform on a complex eukaryotic proteome. The genome of the reference plant *Arabidopsis* was completed over a decade ago (Arabidopsis Genome Initiative, 2000) and now represents one of the most highly curated and annotated genomes in biology. As a consequence, *Arabidopsis* is being utilized as a proving ground for plant synthetic biology approaches, many of which have focused on biomass manipulation for efficient biofuels production (Eudes et al., 2012, 2015). The most recent proteome release (TAIR10) from The Arabidopsis Information Resource (Lamesch et al., 2012) comprises over 27,000 loci and over 35,000 distinct protein products. Protein extractions were performed from a 7-day old *Arabidopsis* cell cultures that have previously been extensively employed for proteomic assays (Parsons et al., 2012). A total protein sample was analyzed in triplicate by UHPLC-QTOFMS using the standard flow proteomics platform. We analyzed the equivalent of ca. 40 μg of digested total protein over a 120 min gradient with MS conditions identical to those used with the *E. coli* samples. An average of 30,575 ± 3,458 (SE) MS/MS spectra were collected over the 2-hour run from the three replicates (Table 1). These numbers are similar to those obtained using the *E.coli* samples and may reflect the upper capacity of the method given the increased peptide complexity that would be expected from a eukaryotic sample.

These *Arabidopsis* datasets were each used to interrogate the most recent *Arabidopsis* protein dataset using the Mascot search engine and matches filtered and integrated using Scaffold. This resulted in an average of 7,532 ± 1,115 (SE) matched spectra from the three replicates and corresponds to about 25% of the total spectra obtained (Table 1). This corresponds to about half the conversion rate observed for the *E.coli* samples and likely reflects the quality of the MS/MS spectra given the overall ion intensities are likely on average considerably lower due to the increased size of the proteome. The average number of proteins identified over the three samples was 1,288 ± 73 (SE) with a total of 1,364 unique proteins identified in all three replicates (Table S2 in Supplementary Material). The number of proteins consistently identified by all three replicates was 1,188 proteins or 87% of the total number (Figure 1B). The minor variation in identifications between these technical replicates demonstrates the reproducibility of the standard flow approach for shotgun proteomics as the method was capable of consistently identify the same proteins in each of the replicates. In an attempt to understand whether the approach is adequately dealing with the increased complexity of this eukaryotic sample, we examined the proportion of unique matched peptides identified in each replicate. The 1,364 unique proteins identified in these samples were matched using 5,419 unique peptides, while the 1,188 proteins identified in all three replicates only required 2,187 (40.4% of the total) unique peptides (Figure 1B). These results indicate that again, as found with the *E. coli* samples, the majority of peptides exclusive to a given replicate are derived from proteins that have already yielded a high scoring peptide match. It is conceivable that the complexity of the eukaryotic sample contributes to many more co-eluting peptides during the 120 min gradient than the *E. coli* sample, resulting in unique peptides selected for MS/MS by the data-dependent acquisition method in each replicate (Figure 1B). Although similar results with regard to unique peptides were also observed with the *E. coli* samples, on average, an *E.coli* protein was identified by 6.8 unique peptides (768 proteins from 5,218 unique peptides) compared to the *Arabidopsis* proteins at 4.0 unique peptides each (1,364 proteins from 5,419 unique peptides). Nearly twice as many proteins were identified in the *Arabidopsis* samples (1,364 proteins compared to 768 proteins), which would be expected given the differences in coding capacity between these species. However, considerably more identified proteins would have been expected given proteome sizes, indicating that the standard flow approach has a more limited capacity to deal with the eukaryotic sample due to issues, such as increased sample complexity at any given point in time, lower overall ion intensity, and dynamic range limitations (as discussed above) of this QTOFMS system.

### Assessing the performance of standard flow LC-MS/MS

**Figure 2:**
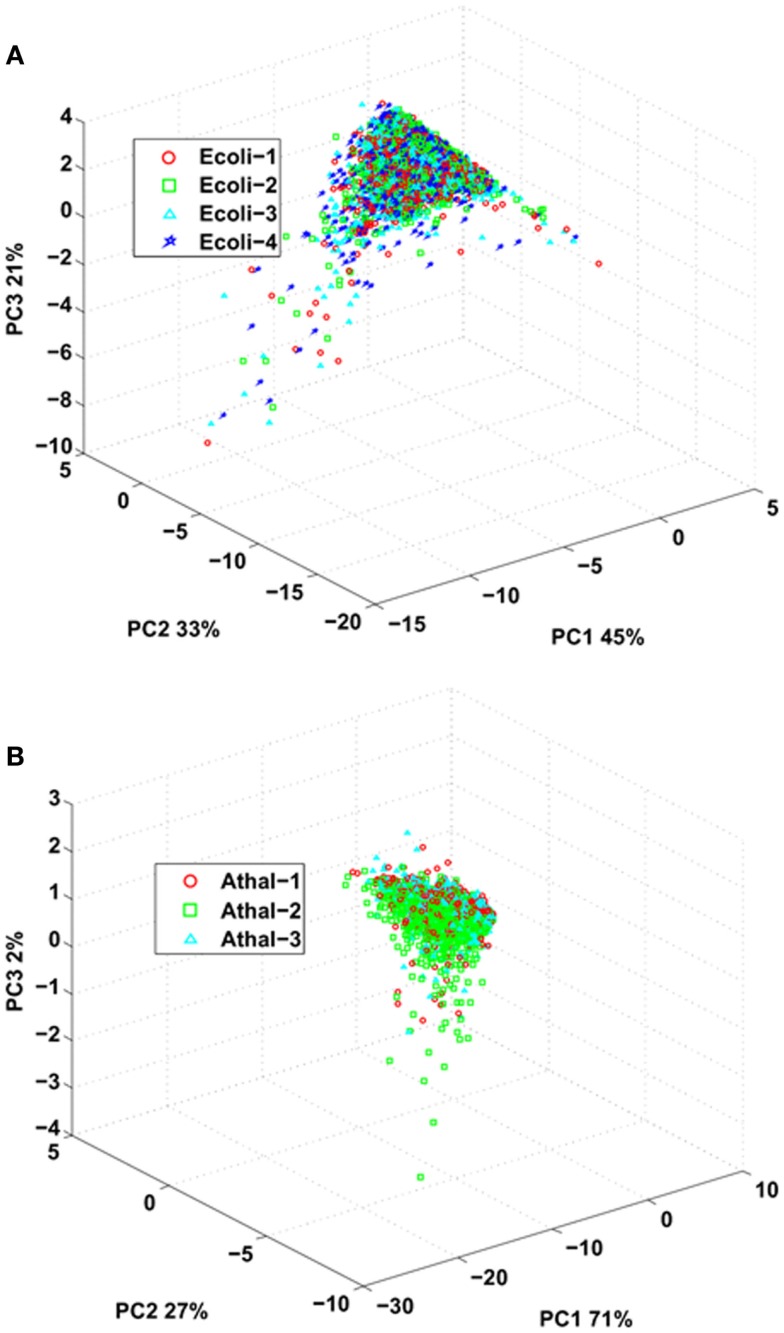
**Principal component analysis of spectral counts, Mascot ions score, and total ion current (derived from MS/MS) from common peptides identified over all replicates from (A) *E. coli* and (B) *Arabidopsis***.

Last, we investigated the reproducibility of the standard flow UHPLC-QTOFMS by comparing parameters of the identified peptides between the replicates for both *E. coli* and *Arabidopsis* samples. The peptide ions score obtained from Mascot after data interrogation can be indicative of the quality of the fragmentation spectra (Perkins et al., 1999), total ion current obtained from the MS/MS spectra can provide information about the intensity of an eluted peptide (Asara et al., 2008) while total spectral count can provide an indication of peptide intensity and spectral complexity (Lundgren et al., 2010). These values can be used as a proxy to assess sample limitations and reproducibility by the LC-MS system. PCA was employed to ascertain whether there were any differences between ion scores, total ion current, and spectral count for peptides identified in all the replicates for either *E. coli* or *Arabidopsis* samples (Figure 2). The analysis demonstrated that none of the replicates for either *E.coli* or *Arabidopsis* could be separated by principal component scores for these attributes, indicating that majority of the differences for either data set can be largely attributed to biological heterogeneity of the sample (Figure 2).

The similarities in ion score, total ion current, and spectral counts can also be observed when analyzing the distributions of CV for the each identified peptides across the replicates (Figure S2 in Supplementary Material). The variations in Mascot ion score and total ion current (derived from MS/MS spectra) for the peptides identified over the replicates were similar for both *E.coli* and *Arabidopsis*. This indicates that from sample to sample, identical ions performed similarly with regard to the intensity of matched peptides (total ion current) as measured by the mass spectrometer. This is further supported by the small variation in Mascot ion scores with ion intensity having a relationship to the quality of the MS/MS spectra and subsequent spectral matching procedures. The variation in total spectrum count was more pronounced between the identified peptides of *E.coli* (0.67) and *Arabidopsis* (0.32), with samples of lower complexity (i.e., *E.coli*) having a larger variation in spectral counts for a peptide across replicates. This could be due to the higher repeat sampling rate that likely occurred during analysis of *E.coli* samples due to the reduced number of distinct ions/peptides in the sample. This conclusion is supported when looking at the average number of spectral counts for a given identified peptide; for *E.coli*, it was 3.74 spectra per peptide while for *Arabidopsis* it was 2.70. The lower average number of spectral counts for a peptide from *Arabidopsis* is likely indicative of the increased sample complexity. Collectively, these data indicate that between the replicates, the quality and intensities of peptides identified across the replicates was similar, indicating that the standard flow approach was not significantly impeding the performance of the mass spectrometer to acquire tandem mass spectra.

## Conclusion

The recent progress of bioenergy research has relied heavily on transformational developments in feedstock characterization and metabolic engineering. Yet, omics methods to characterize and quantify systems of interest have mainly been adapted from the health and clinical fields that have very different research needs (i.e., high sensitivity, deep proteome coverage). We report the application of a standard flow LC-MS/MS approach that is suitable for large numbers of shotgun proteomic experiments where sample abundance is not limiting. The setup is capable of undertaking a rapid analysis of low complexity samples as well as handling highly complex samples by employing extended analysis times. Although its application requires instrumentation with the ability to deal with increased flow rates and shorter peptide elution times, it is apparent that the current generation of tandem mass spectrometers is capable of handling these parameters. While traditional nano-LC-MS/MS approaches are likely to continue to dominate shotgun analyses as they produce a greater number of protein identifications and have the ability to deal with increased sample complexity and dynamic range, the robust nature and simplicity of standard flow coupled to MS makes this approach an attractive alternative for applications where sample throughput and reproducibility are important factors.

## Author Contributions

JH and CP conceived and advised in all aspects of the study. PA supervised all aspects of the study. SG and LC performed experiments. AS analyzed the data. JH and CP interpreted the data and wrote the manuscript. All authors discussed and commented on the manuscript.

## Conflict of Interest Statement

The authors declare that the research was conducted in the absence of any commercial or financial relationships that could be construed as a potential conflict of interest.

## Supplementary Material

The Supplementary Material for this article can be found online at http://journal.frontiersin.org/article/10.3389/fbioe.2015.00044/abstract
