# Supplementary figures and images for: Standard Flow Liquid Chromatography for Shotgun Proteomics in Bioenergy Research
Source: Front Bioeng Biotechnol. 2015 Apr 1;3:44. doi: 10.3389/fbioe.2015.00044 (PMC4381839; doi:10.3389/fbioe.2015.00044)

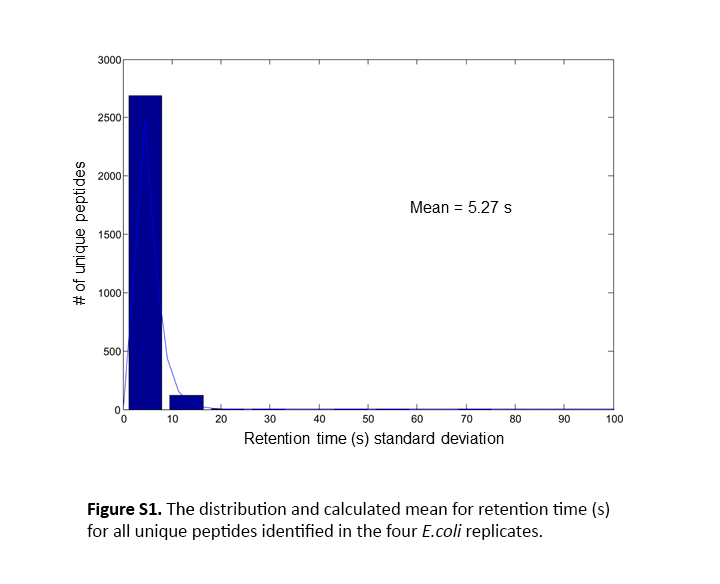

Supplement: Supplementary file 3 [file Image_1.TIF]

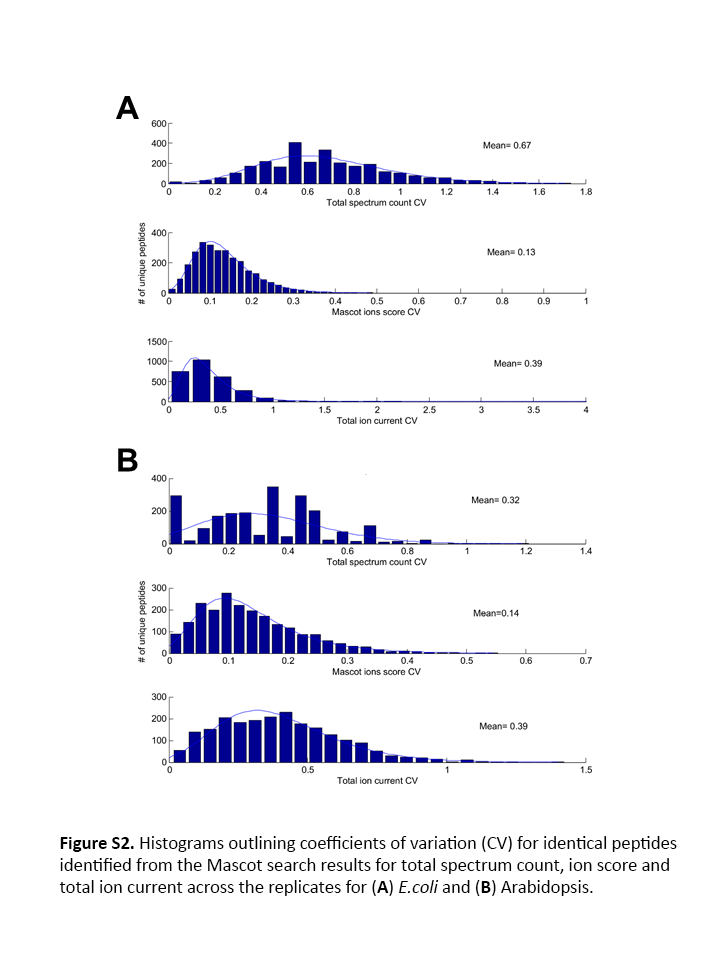

Supplement: Supplementary file 4 [file Image_2.TIF]
